# Dengue Fever: Prognostic Insights From a Complete Blood Count

**DOI:** 10.7759/cureus.11594

**Published:** 2020-11-20

**Authors:** Amogh Ananda Rao, Raaju R U, Siddharth Gosavi, Sanjana Menon

**Affiliations:** 1 Internal Medicine, Jagadguru Jayadeva Murugarajendra Medical College, Davangere, IND

**Keywords:** dengue, lymphocyte, complete blood count, prognosis, mortality, primary care, cost effective

## Abstract

Introduction

Dengue fever is endemic in more than 100 countries. Early indicators of prognosis are vital to reduce the fatality rate associated with dengue fever. The objective of this study is to investigate the value of a complete blood count (CBC) in determining the prognosis of dengue fever.

Methodology

This was a retrospective study of all patients admitted to Chigateri General and Bapuji hospitals, Davangere over two months. Fifty-six patients were included in the study. Medical records were accessed to obtain data on the clinical profile and laboratory investigations.

Results

Thrombocytopenia was the most common hematological feature, in 50 cases (~90%), followed by leukopenia in 43 cases (~76%). The duration of hospital stay ranged from two to seven days. Interestingly, the percentage of lymphocytes in the differential leukocyte count at the time of admission showed a significant negative correlation with the duration of hospital stay (p=0.028). Also, three distinct trends were observed in the sequence of recovery of platelets and white blood cells (WBCs).

Discussion

A repertoire of prognostic indicators have been described to predict the course and outcome of dengue fever: liver enzymes, interleukins 4 and 10, tumor necrosis factor α (TNFα), some proteases, soluble adhesion molecules, the surface area of atypical lymphocytes, high fluorescent lymphocyte counts, immature granulocytes and immature platelet factor (IPF). However, these markers are not routinely employed due to financial constraints and lack of infrastructure.

The percentage of lymphocytes in the differential leukocyte count performed at the time of admission predicted the length of hospital stay. The higher the percentage of lymphocytes, the faster the recovery from dengue and shorter the duration of stay in the hospital. This is particularly important in remote areas with limited laboratory facilities. High-risk patients can be referred to a higher centre before they develop complications of the disease.

Conclusion

The complete blood count can function as an early indicator of prognosis in dengue fever even in areas where sophisticated biomedical infrastructure is lacking. The lymphocyte percentage on admission could significantly predict the length of hospital stay.

## Introduction

Benjamin Rush first described dengue fever in the late eighteenth century [1]. Dengue is derived from a Swahili term, “Ka-dinga pepo” which means a seizure caused by an evil spirit [2]. One of the first infectious diseases to be ascribed to a virus, there are primarily five serotypes in the family of Flaviviridae. Dengue is transmitted by the bite of a mosquito, *Aedes albopictus*. The incubation period is around one week [3].

The affliction with dengue virus has become endemic in more than 100 countries with a higher range of infectivity between August and November. In India, the total economic burden due to dengue in 2018 was USD 27.4 million. The case fatality rate was 2.6%. In 2019, the incidence of dengue cases in India was 157,315, with 16,986 in the state of Karnataka [4].

A single-stranded positive-sense ribonucleic acid (RNA) virus, belonging to the family Flaviviridae, is the cause of dengue fever. The viral genome consists of 11,000 bases, and codes for three structural proteins (C, prM and E) that form the virus particle and seven non-structural proteins (NS1, NS2a, NS2b, NS3, NS4a, NS4b and NS5).

Patients with dengue must be evaluated thoroughly in a manner to prevent progression to severe disease. The febrile phase consists of high-grade fever, headache, vomiting, myalgia, arthralgia and transient macular rash rarely. Recovery without complications is not uncommon in this phase. Anorexia, nausea, vomiting, abdominal pain, diarrhoea, haemorrhagic manifestations and respiratory symptoms may occur. Examination reveals conjunctival injection, pharyngeal erythema, lymphadenopathy, hepatomegaly, facial puffiness and petechiae [5,6,7].

A small proportion of patients develop a systemic vascular syndrome characterised by bleeding, shock, encephalopathy, seizures and organ impairment. This is called the critical phase and lasts 24 to 48 hours. Hypotension usually occurs in this phase. Moderate to severe thrombocytopenia is observed with a temporary increase in the activated partial thromboplastin time and reduced fibrinogen levels. Hepatic failure, central nervous system involvement, myocarditis, acute kidney injury can occur in this phase [8-13].

The convalescent phase starts only when there is a resolution of haemorrhage and plasma, stabilization of vital signs and reabsorption of fluid accumulation. This phase lasts two to four days.

Reverse transcriptase-polymerase chain reaction assay positive during the first five days of illness or detection of viral antigen non-structural protein 1 positive during the initial seven days of illness are the standard for diagnosis [14-16].

In terms of blood investigations, leucopenia is observed usually in the course of dengue fever. Thrombocytopenia is considered a predictor of dengue hemorrhagic fever (DHF). Haemoconcentration is also a common finding due to the concentration of plasma fluid leakage, causing an increase in the haemoglobin weight in a unit volume of blood [17].

Mortality due to dengue fever occurs mainly in cases of severe dengue with complications, more frequently in patients with underlying comorbidities. The social determinants are increasing age, poor health-seeking behavior, low income and less education. [18]

Therefore, early indicators of prognosis, available and accessible even in resource-limited settings offering primary healthcare, are vital to reduce the fatality rate associated with dengue fever. The primary objective of this study is to investigate the value of a complete blood count (CBC) in determining the prognosis of dengue fever. The second objective is to observe the trends of recovery of white blood cells (WBCs) and platelets in dengue fever.

## Materials and methods

**Figure 1:**
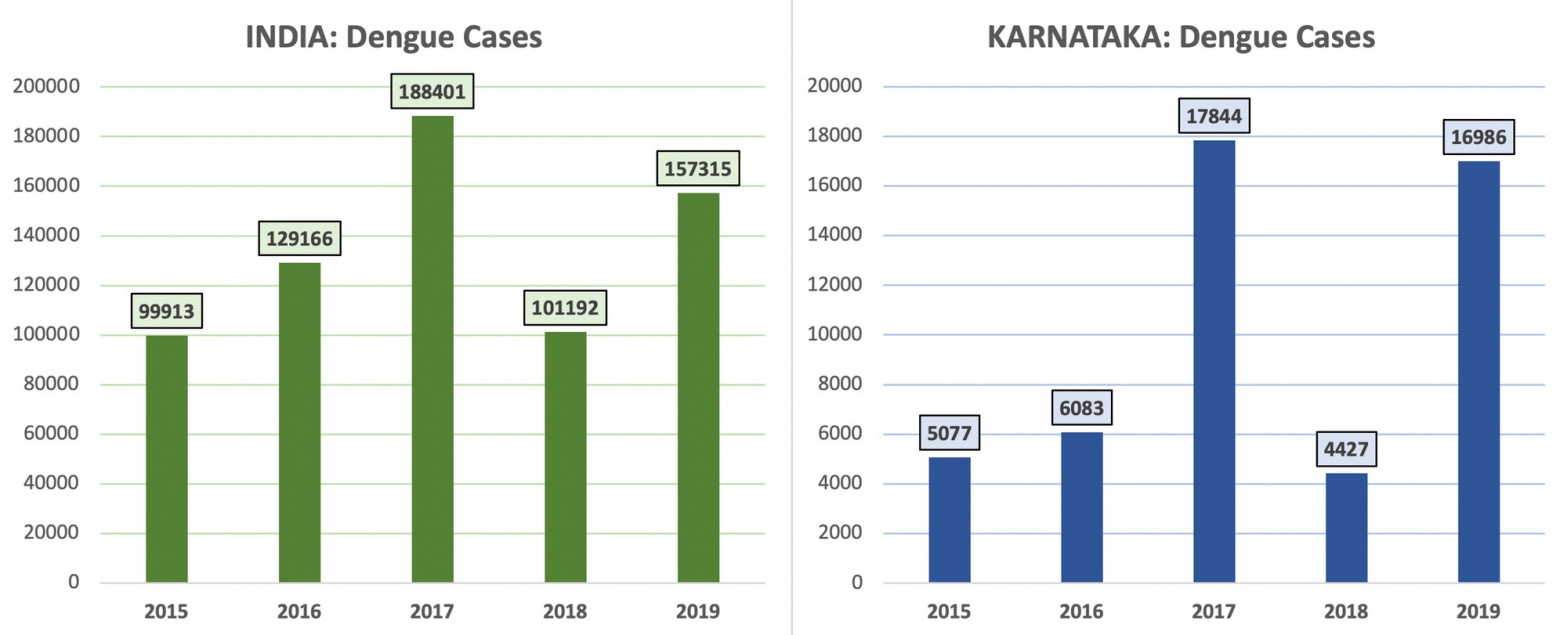
Incidence of Dengue Fever in India and Karnataka

This was a retrospective study conducted for two months, July and August at Chigateri General and Bapuji hospitals, Davangere, the central province in the state of Karnataka. The incidence of dengue fever in Karnataka is shown in Figure 1 [4].

**Figure 2:**
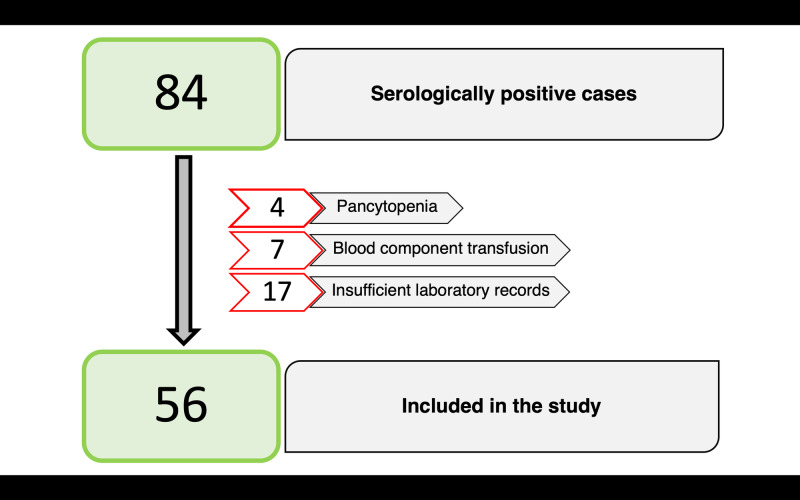
Sample size after application of exclusion criteria

The study was started after obtaining a clearance from the Institutional Ethics Committee. All inpatients who tested positive for the non-structural antigen 1 (NS1) or the immunoglobulin M (IgM) antibody were enrolled in the study. The exclusion criteria were patients with pancytopenia secondary to other causes, blood component transfusion, chronic liver disease, haematological disease, and administration of immunosuppressive drugs, including steroids. Nonetheless, a sizeable fraction of the sample could not be included in the analysis due to non-availability or insufficient medical or laboratory records. The final sample contained 56 patients (Figure 2).

The demographic details, presenting complaints, the clinical diagnosis and the duration of hospital stay of the patient were documented. Medical records were accessed to obtain data from the CBCs. Only patients with a minimum of five CBCs were included in the study. The frequency of tests in each case was based on the clinician’s discretion in accordance with the patient’s condition. Therefore, we use the term, ‘reading’ to denote successive CBCs. The haemoglobin concentration, haematocrit, platelet count, total leukocyte count and differential leukocyte count were all recorded and tabulated. Data analysis was performed on Microsoft Excel for Mac, version 16.31. Demographic and clinical data were presented as descriptive statistics. The patterns of recovery of platelets and WBCs were studied. Parameters in the CBC and clinical characteristics were analysed using linear and multiple regression techniques. A p-value of less than 0.05 was considered statistically significant.

Thrombocytopenia was defined as a platelet count of less than 150,000/mm3, and leukocytopenia was defined as a total WBC count below 4000 cells/mm3.

## Results

**Figure 3:**
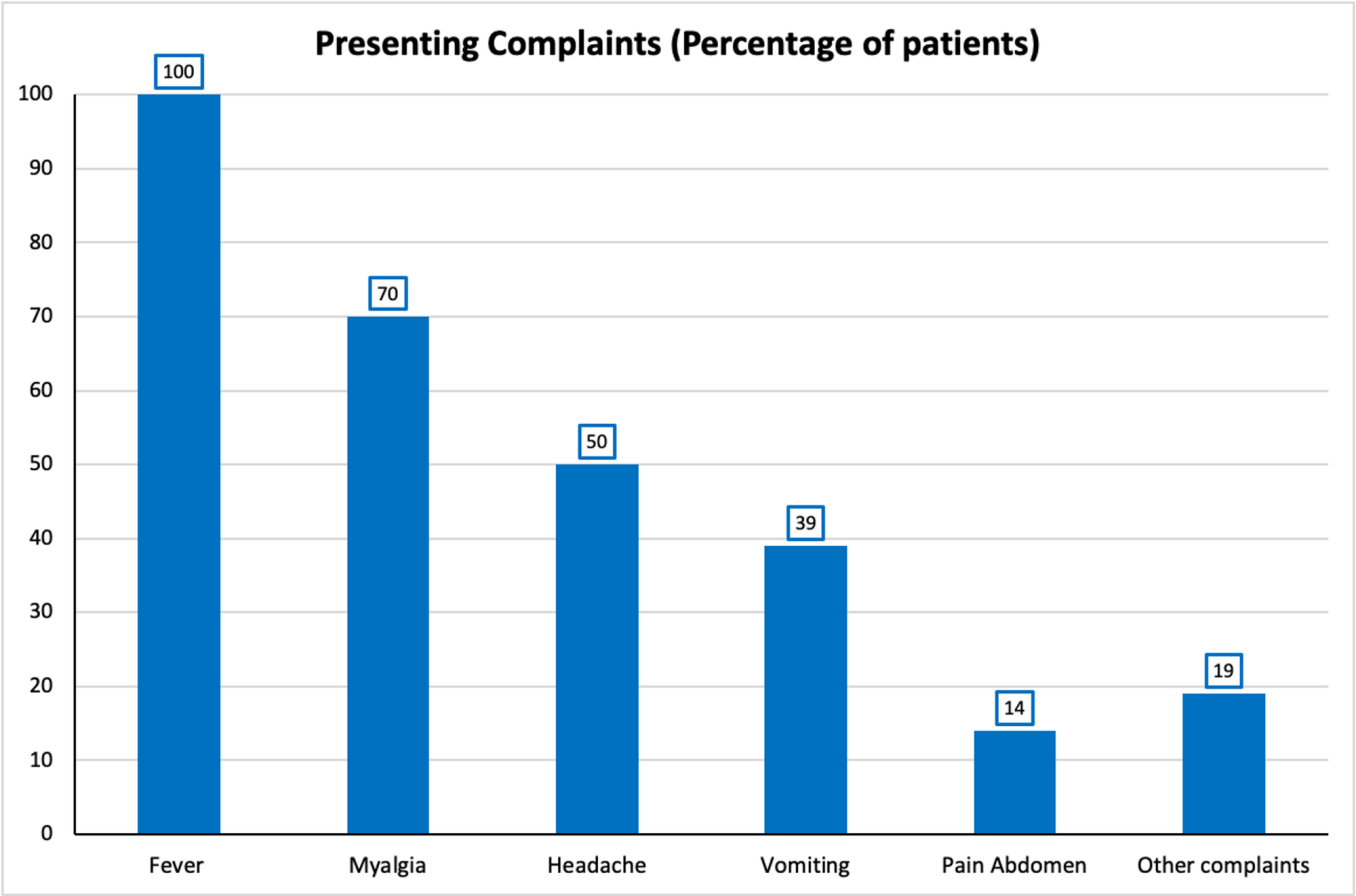
Presenting complaints of patients

**Figure 4:**
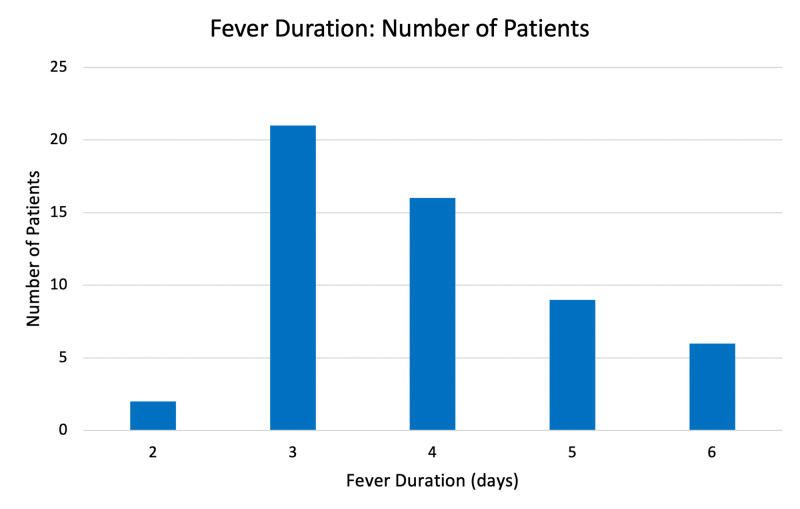
Duration of Fever

Of the 56 patients enrolled in the study, 35 were male, and 21 were female. The mean age of the sample was 28.2 years, ranging from 11 to 63 years. All patients presented with a history of fever ranging from two to six days, along with other complaints: myalgia, headache, vomiting, pain abdomen, and urinary symptoms (Figure 3, 4). The duration of hospital stay ranged from two to seven days.

Thrombocytopenia was the most common hematological feature, in 50 cases (~90%), followed by leukopenia in 43 cases (~76%).

**Figure 5:**
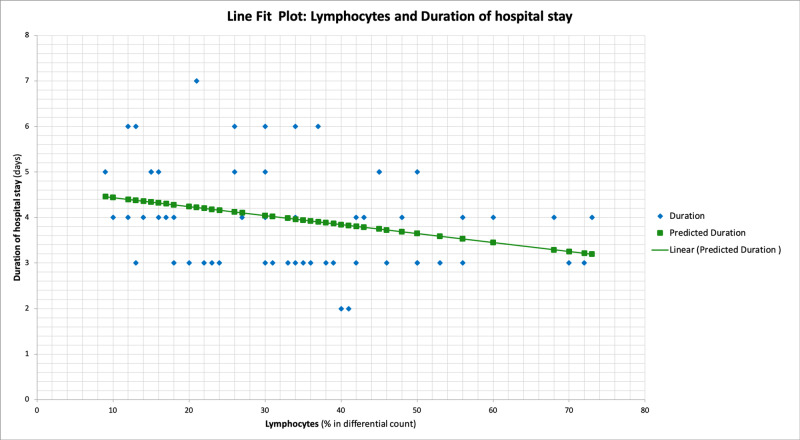
Line fit plot showing the association of Lymphocyte percentage in the differential count and the duration of hospital stay

Interestingly, the percentage of lymphocytes in the differential leukocyte count at the time of admission showed a significant negative correlation with the duration of hospital stay (p=0.028) (Figure 5).

**Figure 6:**
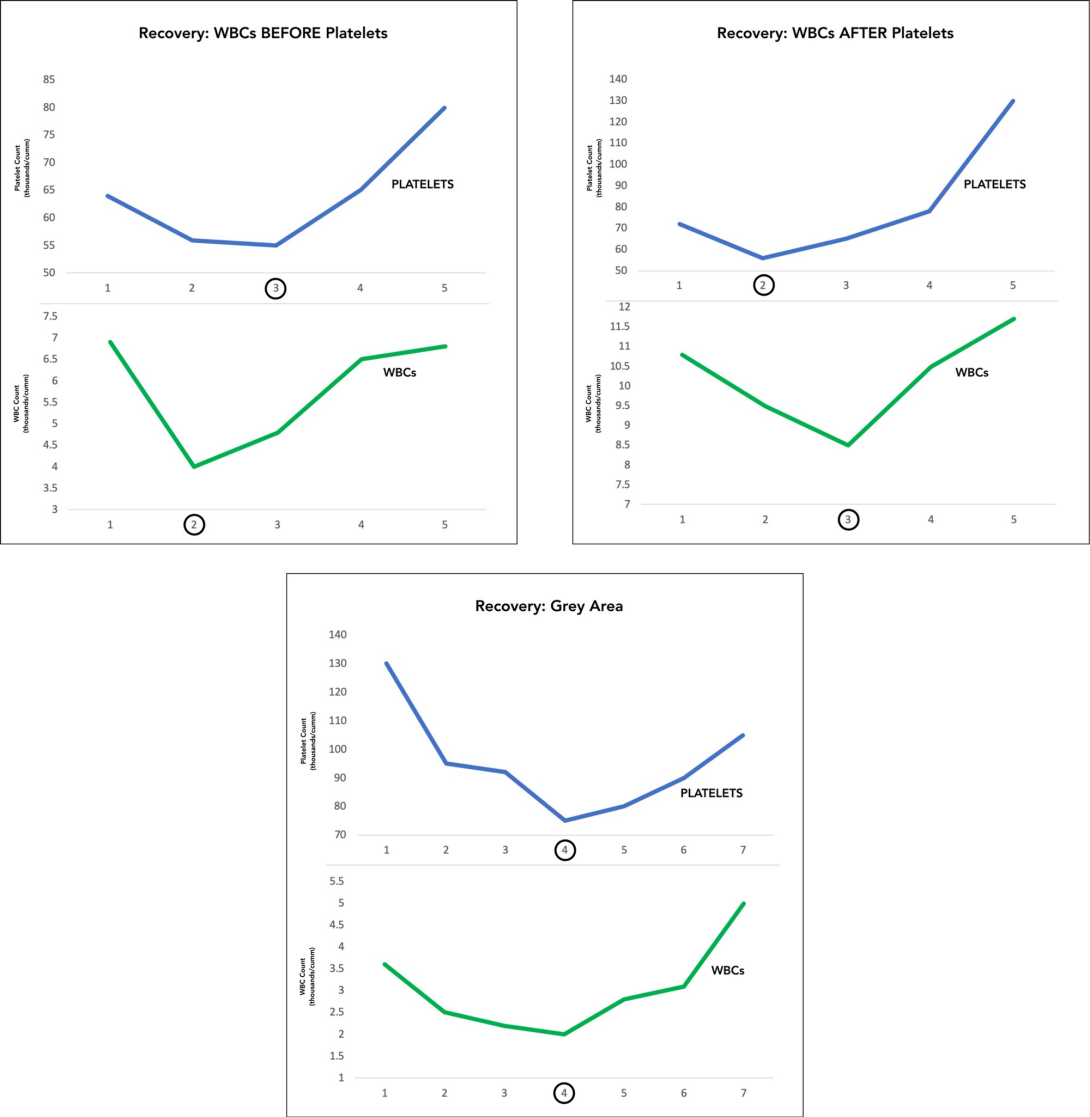
Trends in the recovery of white blood cells (WBCs) and platelets

**Figure 7:**
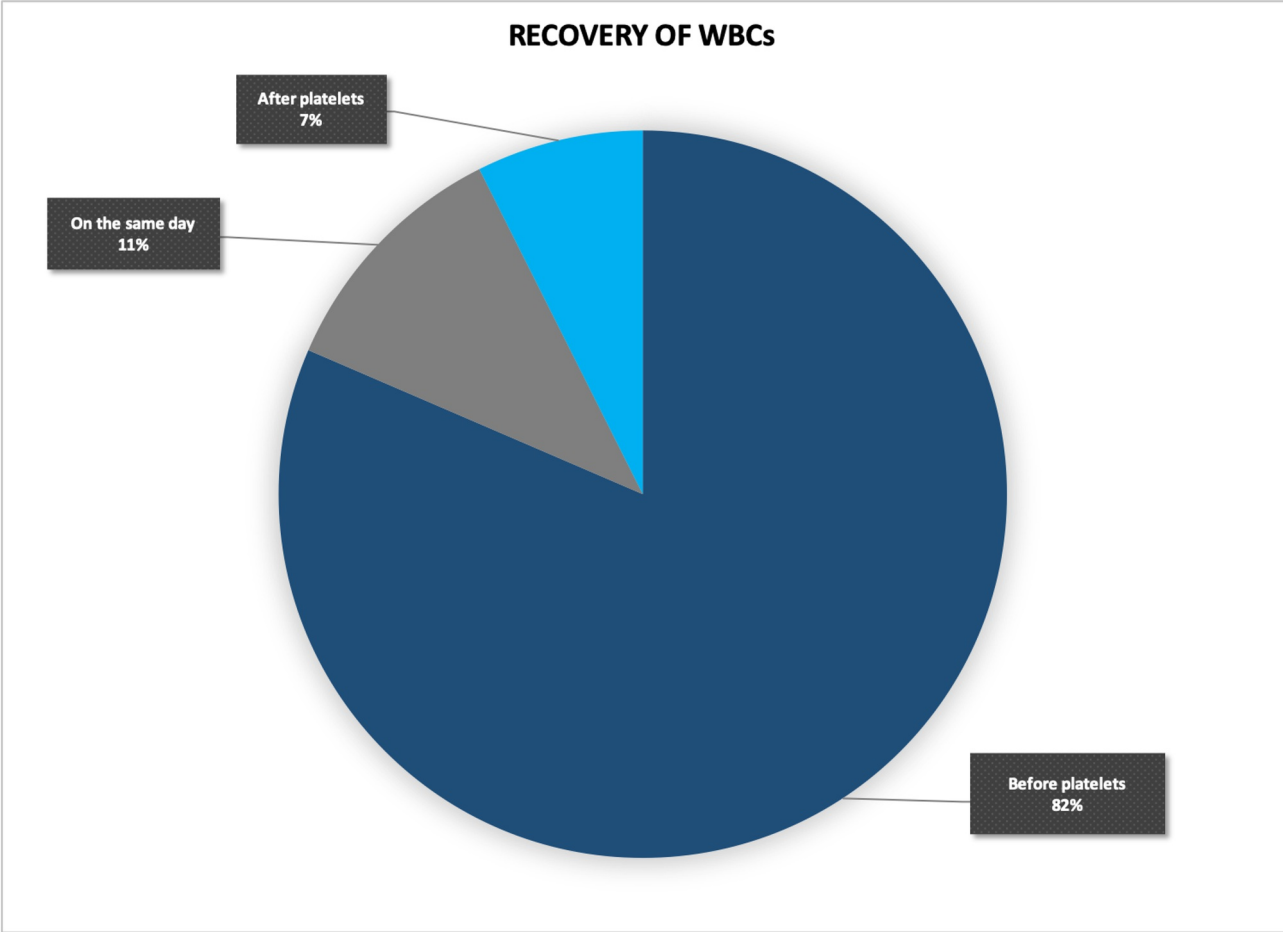
White blood cell (WBC) recovery in relation to platelets

Three distinct trends were observed in the sequence of recovery of platelets and WBCs (Figure 6). Firstly, the resurgence of WBCs in 82% of the cases foreshowed the recovery of platelets. Secondly, in 7% of the cases, platelets recovered earlier than the WBCs. Thirdly, in 11% of the cases, both the cell lineages showed a recovery in the same reading (Figure 7). However, this is a grey area considering the ambiguity in the order of recovery. This uncertainty can be overcome by performing CBCs more frequently, to capture the real winner in the race of resurgence. In the following figure, one case representing each trend has been shown. The three trends showed no significant association with a clinical parameter. No other significant correlation was observed amongst the clinical and laboratory parameters.

## Discussion

In our study, the most common hematological feature was thrombocytopenia, followed by leukopenia and an increase in the hematocrit. Leukocytosis was relatively uncommon, present only in cases of a probable superadded bacterial infection or sepsis. Lymphocytosis was observed from the third to the sixth day of fever. However, in a study conducted by Raimunda et al., leukocytosis was observed in patients with classical dengue in the initial days of the disease. Lymphocytosis was more pronounced from the fourth to the sixth day [19].

The percentage of lymphocytes in the differential leukocyte count performed at the time of admission turned out to be a predictor of the length of hospital stay. The higher the percentage of lymphocytes, the faster the recovery from dengue and shorter the duration of stay in the hospital. This is particularly useful in remote areas with limited laboratory facilities. Patients with low lymphocytes and co-morbidities can be referred to a higher centre before they develop complications of the disease.

One of the theories implicated in the causation of thrombocytopenia is the viral induction of bone marrow hypoplasia by affecting the bone marrow progenitor cells. A significant derangement in the plasma-kinin system, disseminated intravascular coagulation, increased apoptosis resulting in platelet destruction, lysis by the complement system and the formation of anti-platelet antibodies are crucial factors [20].

Chaloemwong et al. reported leukopenia from day two, reaching the minimum on day five followed by a gradual recovery. This is believed to be due to destruction of myeloid progenitor cells with hypocellular bone marrow in the first seven days of fever. Atypical lymphocytes appeared from day five to day nine, hitting the peak on day seven. Monocytosis was observed from day one to day four and eosinophilia occurred from day nine to day ten.

A repertoire of prognostic indicators has been described to predict the course and outcome of dengue fever. An increase in aspartate aminotransferase (AST) occurred in the beginning and remained stable in all clinical forms. Alanine aminotransferase (ALT) started worsening on the seventh day of the disease. Nonetheless, an early increase in ALT has been associated with severe dengue [21]. Interleukins 4 and 10, which play a significant role in the pathogenesis of dengue fever, are used as markers of severe dengue [22]. Tumor necrosis factor α (TNFα) induces apoptosis of the endothelial cells and shows promise as a biomarker of severe dengue. Some proteases, soluble adhesion molecules and metabolites are possible indicators of disease severity. However, there is insufficient evidence for routine employability. Flow cytometry quantitates the surface area of atypical lymphocytes, high fluorescent lymphocyte counts, immature granulocytes and immature platelet factor (IPF) [23]. Although these markers provide considerable accuracy, they are not routinely feasible due to financial constraints and lack of infrastructure. Therefore, even in a resource-limited setting, a CBC can be used to gauge the prognosis of the patient.

Limitations

The primary limitation of the study was the small sample size. Only inpatients were enrolled in the study and thus the recovery patterns in patients with a milder form of dengue were not studied. The interval between consecutive CBCs was not consistent in all patients as it was based on the clinician's discretion.

## Conclusions

Prognostic indicators of dengue fever generally rely on advanced investigations which may not be available in centres providing primary health care. The complete blood count can function as an early indicator of prognosis in dengue fever even in areas where sophisticated biomedical infrastructure is lacking. The lymphocyte percentage on admission could significantly predict the length of hospital stay. Nonetheless, larger studies involving multiple centres are necessary to validate the findings.

## References

[1] Barnett R (2017). Dengue. Lancet.

[2] Packard RM (2016). “Break-Bone” fever in Philadelphia, 1780: reflections on the history of disease. Bull Hist Med.

[3] Normile D (2013). Surprising new dengue virus throws a spanner in disease control efforts. Science.

[4] (2020). Dengue Cases in India. https://main.mohfw.gov.in/media/disease-alerts/dengue.

[5] Fukusumi M, Arashiro T, Arima Y (2016). Dengue sentinel traveler surveillance: monthly and yearly notification trends among Japanese travelers, 2006-2014. PLoS Negl Trop Dis.

[6] Trofa AF, DeFraites RF, Smoak BL (1997). Dengue fever in US military personnel in Haiti. JAMA.

[7] Leder K, Torresi J, Brownstein JS (2013). Travel-associated illness trends and clusters, 2000-2010. Emerg Infect Dis.

[8] Kalayanarooj S, Vaughn DW, Nimmannitya S (1997). Early clinical and laboratory indicators of acute dengue illness. J Infect Dis.

[9] Chhour YM, Ruble G, Hong R (2002). Hospital-based diagnosis of hemorrhagic fever, encephalitis, and hepatitis in Cambodian children. Emerg Infect Dis.

[10] Solomon T, Dung NM, Vaughn DW (2000). Neurological manifestations of dengue infection. Lancet.

[11] Carod-Artal FJ, Wichmann O, Farrar J, Gascón J (2013). Neurological complications of dengue virus infection. Lancet Neurol.

[12] Neeraja M, Iakshmi V, Teja VD (2014). Unusual and rare manifestations of dengue during a dengue outbreak in a tertiary care hospital in South India. Arch Virol.

[13] Verma R, Sahu R, Holla V (2014). Neurological manifestations of dengue infection: a review. J Neurol Sci.

[14] Guzman MG, Jaenisch T, Gaczkowski R (2010). Multi-country evaluation of the sensitivity and specificity of two commercially-available NS1 ELISA assays for dengue diagnosis. PLoS Negl Trop Dis.

[15] Hunsperger EA, Muñoz-Jordán J, Beltran M (2016). Performance of dengue diagnostic tests in a single-specimen diagnostic algorithm. J Infect Dis.

[16] Huits R, Soentjens P, Maniewski-Kelner U (2017). Clinical utility of the nonstructural 1 antigen rapid diagnostic test in the management of dengue in returning travelers with fever. Open Forum Infect Dis.

[17] Heilman JM, De Wolff J, Beards GM, Basden BJ (2014). Dengue fever: a Wikipedia clinical review. Open Med.

[18] Carabali M, Hernandez LM, Arauz MJ, Villar LA, Ridde V (2015). Why are people with dengue dying? A scoping review of determinants for dengue mortality. BMC Infect Dis.

[19] Azin FR, Gonçalves RP, Pitombeira MH, Lima DM, Branco IC Dengue: profile of hematological and biochemical dynamics. Rev Bras Hematol Hemoter.

[20] de Azeredo EL, Monteiro RQ, de-Oliveira Pinto LM (2015). Thrombocytopenia in dengue: Interrelationship between virus and the imbalance between coagulation and fibrinolysis and inflammatory mediators. Mediators Inflamm.

[21] Samanta J, Sharma V (2015). Dengue and its effects on liver. World J Clin Cases.

[22] Abhishek KS, Chakravarti A, Baveja CP, Kumar N, Siddiqui O, Kumar S (2017). Association of interleukin-2, -4 and -10 with dengue severity. Indian J Pathol Microbiol.

[23] Chaloemwong J, Tantiworawit A, Rattanathammethee T, Hantrakool S, Chai-Adisaksopha C, Rattarittamrong E, Norasetthada L (2018). Useful clinical features and hematological parameters for the diagnosis of dengue infection in patients with acute febrile illness: a retrospective study. BMC Hematol.

